# Chemical Composition, Antioxidant Activity, and Sensory Characterization of Commercial Pomegranate Juices

**DOI:** 10.3390/antiox10091381

**Published:** 2021-08-29

**Authors:** Sonia Esposto, Gianluca Veneziani, Agnese Taticchi, Stefania Urbani, Roberto Selvaggini, Beatrice Sordini, Luigi Daidone, Giacomo Gironi, Maurizio Servili

**Affiliations:** Department of Agricultural, Food and Environmental Sciences, University of Perugia, Via San Costanzo s.n.c., 06126 Perugia, Italy; sonia.esposto@unipg.it (S.E.); gianluca.veneziani@unipg.it (G.V.); agnese.taticchi@unipg.it (A.T.); stefania.urbani99@gmail.com (S.U.); roberto.selvaggini@unipg.it (R.S.); luigi.daidone@unipg.it (L.D.); giacomo.gironi@studenti.unipg.it (G.G.); maurizio.servili@unipg.it (M.S.)

**Keywords:** commercial pomegranate juices, bioactive compounds, ellagitannins, anthocyanins, antioxidant activity, organic acids, volatile compounds, sensory evaluation

## Abstract

We undertook a qualitative and quantitative assessment of the bioactive compounds, volatile substances, sensory profile, and antioxidant activity of eight different commercial pomegranate juices (PJs) differing by cultivation area, processing (from concentrate (CPJ) or not (NCPJ)), and microbial stabilization. Punicalins were the main ellagitannins, whereas the predominant anthocyanin was cyanidin 3,5-diglucoside, followed by cyanidin 3-glucoside. Total phenols, tannins, hydrolyzable tannins, and anthocyanins in the investigated juices ranged from 1379.9 to 3748.8 mg gallic acid equivalent (GAE)/L, 394.8 to 895.2 mg GAE/L, 150.8 to 2374.2 mg ellagic acid/L, and 0 to 281 mg cyanidin 3-glucoside/L, respectively. Antioxidant activity, determined by DPPH^•^, FRAP, and ABTS, was positively correlated with the total phenolic compounds and hydrolyzable tannins. Alcohols, acids, and furans were the volatile groups that best described the differences between juices. The most abundant volatile compound was 3-Furfural (8.6–879.9 µg/L) in those juices subjected to the concentration process and to high-temperature short-time (HTST) treatment, whereas it was not present in juice subjected to high-pressure processing (HPP). Processing also affected the juices’ sensory attributes: NCPJs were characterized by ‘red–purple’, ‘pomegranate fruit’, ‘fresh fruit’, and ‘overall intensity’ notes, while in CPJs these notes were not revealed or were masked by those related to heat treatment. Our results provide useful information on the importance of extraction methods and on the overall quality of PJ for the food industry.

## 1. Introduction

Pomegranate cultivation around the world has reached more than 300,000 Ha, with an estimated production of over 4,500,000 t/year [1].

The increasing popularity of this fruit and its products (the juice in particular) is due to it being a valuable source of bioactive compounds, represented by different classes of polyphenols [2,3].

The main polyphenolic substances, characterized by well-known antioxidant properties [4,5,6], are hydroxybenzoic and hydroxycinnamic acids, flavonoids, leucoanthocyanidins, proanthocyanidins, anthocyanins, and condensed and hydrolyzable tannins [7]. In this last chemical class, the most important phenolic substances, in terms of antioxidant and health importance, are α and β punicalagin, α and β punicalin, pedunculagin, and garantin A and B [8]. Their health benefits have already been demonstrated, and more recent research has shown a reduction in the levels of erythropoietin in the serum of patients with type II diabetes [9]; a decrease in the levels of the systolic and diastolic blood pressure and total and LDL cholesterol in diabetic patients [10]; a decrease in blood pressure, creatinine levels, muscle damage parameters, and C-reactive protein in middle-aged athletic men [11]; and a reduction in the blood levels of insulin, glucose, triglycerides, total cholesterol, and LDL in overweight or obese subjects [7].

In addition to the ‘in vivo’ studies, ‘in vitro’ research has focused on the effect of α and β punicalagin, demonstrating their antiproliferative activity in prostate and lung cancer cells, thyroid papilloma, renal carcinoma, and other forms of breast and brain tumors [7,12,13,14].

Beyond the health properties, PJ is also appreciated by consumers for its sensory characteristics due to volatile substances (alcohols, aldehydes, esters, and terpenes, responsible for the typical odour) and organic acids and sugars (responsible for the characteristic acidity and sweetness, respectively). Furthermore, interesting is the contribution to PJ sensory properties of polyphenols, including anthocyanins and tannins, which are positively correlated with red colour intensity and taste and tactile sensations (such as light bitterness and astringency, in particular), respectively [15].

The growing demand for these health-promoting products has driven researchers to identify and select pomegranate cultivars with high yield and with potential sensory and physicochemical characteristics suitable for pomegranate juice industrial processing [5,16]. Nowadays, the ‘Wonderful’ cultivar is most used for PJ production, although there are over 500 known varieties and 200 accessions with different chemical compositions whose germplasm has not yet been fully studied and exploited [8].

The genetic and geographic origin, climatic conditions, ripening stage, agricultural practices, and juice extraction and microbial stabilization methods affect the chemical composition of the PJ [17,18,19,20,21,22] and, therefore, its health properties and sensory qualities [23,24,25,26,27,28,29,30].

Mphahlele et al. [31], comparing various methods of extraction (crushed arils, smoothed arils, whole fruit, and fruit cut in half), did not observe a statistically significant difference in the content of soluble solids; on the contrary, the phenolic compounds and the tannins were higher for fruits cut in half (shredded arils: 138.36 mg GAE/100 mL, whole fruit: 185.37 mg GAE/100 mL, intact arils: 215.21 mg GAE/100 mL, half: 289.94 mg GAE/100 mL). Furthermore, the juice was found to be richest in terms of volatile substances and therefore had a more intense aroma by grinding the arils than that obtained by other extraction methods [31,32,33].

With regard to the microbial stabilization, the most used techniques are based on heat transfer by pasteurization or sterilization and creating a juice that can be refrigerated or stored at room temperature. Microbial stabilization, in particular, has a high impact on the PJ quality, as demonstrated by several authors, with a significant reduction in anthocyanin [23], red colour, ascorbic acid content [34,35], thiamine (Vit. B1), biotin (Vit. B8), pantothenic acid (Vit. B5), pyridoxine (Vit. B6), and riboflavin (Vit. B2) [35], and a significant increase in browning colour [34] with respect to the original fresh juice. However, among all the heat treatments, flash pasteurization was revealed to be the best method for preserving the health and sensory properties of the juice [35].

With mild microbial stabilization, Feng et al. [36] observed a higher number of phenolic compounds (due to the high pressure, responsible for the breakdown of cell residues) and higher ascorbic acid content but a reduced refrigerated shelf life in an HPP-treated juice compared to a pasteurized one. Guo et al. [37], comparing the microbial and chemical effects of pasteurization treatment (105 °C for 5 s) with PEF (pulsed electric field, energies of 35 kV/cm and 38 kV/cm with 2000 pulsations per second of 1 µs each) and a fresh untreated juice, demonstrated an adequate shelf life of the product treated by PEF at 4 °C up to 12 weeks, and non-statistically significant differences in the polyphenolic content as well as colour parameters when compared with the fresh product.

Very often, commercial PJs have been obtained by diluting and stabilizing concentrated fruit juices, which, in fact, represent the main half-processed product in the PJ industry [33]. Indeed, concentration (in general, not lower than 50% of the original water) allows us to reduce the transport and storage costs as well as to prolong the juice shelf-life due to the significant reduction in water activity. Concentration by evaporation is the most used technique, even if it might be responsible for antioxidant and aromatic substance loss and a general increase in ’cooked’ aroma and ’brown’ colour [38], in particular if it is carried out at a normal atmospheric pressure and therefore at 100 °C. Concentration under a vacuum atmosphere, reaching a boiling point at 40–65 °C, allows us to significantly (*p* < 0.05) reduce the juice quality loss [39]. Concentration by microwaves also leads to a reduction in anthocyanins, colour, and antioxidant activity [40]. “Cold” methods such as cryoconcentration and membrane filtration were also tested and shown to have positive effects on the concentrated juice quality. In particular, cryoconcentration allows us to maintain the chemical–sensorial characteristics of the juice in terms of ascorbic acid, total polyphenols, anthocyanins, antioxidant activity, and sensory properties, which remain closer to those of fresh juice [41,42,43,44]. Reverse osmosis (RO) is widely used as a pretreatment of the juice before concentration by evaporation or by cryoconcentration. Few studies have been done on this method. RO with modified polyamide membranes reaches a total soluble solids concentration of 18 °Brix and a permeate flow at the end of the process of 11.3 kg m^−2^ h^−1^ [45,46]. Osmotic distillation (OD) is another cold concentration technique that allows us to create concentrations in solutes that are much higher than those achievable with RO (around 65–70 °Brix) while maintaining the sensory characteristics of fresh juice [46]. Rehman et al. [47] compared the performance of two different membranes in the OD process for the concentration of PJ. A polytetrafluoroethylene (PTFE) membrane showing higher evaporation fluxes allowed them to reach a higher juice concentration (41 °Brix) in a processing time of 24 h with respect to the polyvinylidene fluoride (PVDF) membrane (18 °Brix). The PTFE membrane turned out to be more effective at preserving in the OD concentrate the quality parameters of PJ such as total acidity, total phenolic compounds, total flavonoid content, and total antioxidant activity because wetting did not occur. [47]. However, data in the literature to date have been obtained by comparing PJs produced at a pilot plant scale, which usually do not correspond to the parameters and quantities managed in an industrial plant.

Very little research is available on the characterization of commercial PJs purchasable at a supermarket or online, which are, nowadays, the most important and useful purchasing methods for consumers [48].

Specifically, one of the consequences of COVID-19 has been e-commerce diffusion, in particular for foods and beverages, the quality of which has still not been adequately studied [48].

This paper reports, for the first time, on the evaluation of the overall quality of commercial PJs available in stores and online, differing according to the fruit cultivar and cultivation area, as well as the techniques employed for their safe distribution to consumers.

Specifically, this is the first study to compare instrumental data with sensory data, giving a broad and complete picture of the impact of different several factors on PJ quality.

## 2. Materials and Methods

### 2.1. Reagents

Cyanidin-3-O-glucoside, gallic, and ellagic acid were purchased from Extransynthèse (Genay, France). 2,4,6-Tripyridyl-s-triazine (TPTZ), 6-hydroxy-2,5,7,8-tetramethyl-chroman-2-carboxylic acid (Trolox), 2,2-Diphenyl-1-picrilidrazil (DPPH^•^), 2,2′-Azinobis (3-ethylbenzothiazoline-6-sulfonic acid), and diammonium salt (ABTS) were purchased from Sigma-Aldrich (Milan, Italy). Phosphotungstic phosphomolybdic acid (Folin–Ciocalteu’s reagent), sodium carbonate anhydrous, formic acid, phosphoric acid, hydrochloric acid, methanol, ethanol, and acetonitrile were supplied by VWR (Milan, Italy). HPLC-grade methanol and water for HPLC-MS were supplied by Carlo Erba (Milan, Italy). 4-Methyl-2-pentanol, as an internal standard of PJ volatile compounds, was purchased from Sigma-Aldrich (Milan, Italy).

### 2.2. PJs

Eight 100% PJs were selected, purchased at a store or online, and used for the experiments. The samples were as follows: ICPJ1 (Internet, from concentrate pomegranate juice), INCPJ2 (Internet, not from concentrate pomegranate juice), SNCPJ3 (Store, not from concentrate pomegranate juice), INCPJ4 (Internet, not from concentrate pomegranate juice), SCPJ5 (Store, from concentrate pomegranate juice), SCPJ6 (Store, from concentrate pomegranate juice), ICPJ7 (Internet, from concentrate pomegranate juice), and INCPJ8 (Internet, not from concentrate pomegranate juice). In Table 1, the fruit origin, agronomic, and technological characteristics are reported. Nutritional and energetic values are also reported in Table S1.

### 2.3. Determination of Total Phenolic Compounds and Tannins

The phenolic composition of PJs was evaluated by colorimetric methods using a Cary 100 Scan UV-Visible spectrophotometer (Varian, Walnut Creek, CA, USA). The polyphenols were determined with the Folin–Ciocalteu reagent, according to the Official Method of Analysis, and expressed as mg/L of gallic acid [49]. The analysis of the total tannins was performed by their precipitation with methylcellulose, according to the method proposed by Montedoro and Fantozzi [50].

### 2.4. Determination of Ellagitannins and Their Precursors

PJ was filtered with cellulose acetate (CA) 0.45 μm syringe filters (Whatman, Clifton, NJ, USA) and injected into HPLC. The chromatographic separation of the phenols was carried out in the reverse phase using a Superpher 100 RP-18 column, 4 mm i.d × 250 mm with an internal particle diameter of 5 µm (Agilent Technologies, Palo Alto, CA, USA). The injected volume was 20 µL, and the eluent flow was 1.0 mL/min using a mixture of water/phosphoric acid (99.5:0.5 *v*/*v*) (solvent A) and methanol/acetonitrile (1:1 *v*/*v*) (solvent B). The gradient was varied as follows: 95% solvent A and 5% solvent B, 70% solvent A and 30% solvent B, for 25 min, 62% solvent A and 38% solvent B for 10 min, 55% solvent A and 45% solvent B for 10 min, 48% solvent A and 52% solvent B for 5 min, 0% solvent A and 100% solvent B for 5 min, and return to initial conditions for 5 min. The total chromatographic run time was 60 min. For the determination of phenols, the diode array detector (DAD) was set to the wavelengths of 280 nm and 360 nm. The quantitative evaluation was carried out using the response factor calculated for the ellagic acid.

The identification of ellagitannins and their precursors was carried out by UHPLC-DAD-Q-TOF/MS analysis (Table S2) using an ultra-high-performance liquid chromatography system (UHPLC, Agilent Technologies, mod. 1260 Infinity) consisting of a degasser, binary pump, autosampler, column thermostating oven, and diode array detector, all coupled to a quadrupole–time-of-flight (Q-TOF) mass spectrometer with an electrospray ionization source (Dual ESI, model Agilent 6530 Accurate-Mass Q-TOF LC/MS, Agilent Technologies, Palo Alto, CA, USA). The column used was a Zorbax Eclipse Plus C18 100 mm × 2.1 mm, 1.8 μm (Agilent Technologies, Palo Alto, CA, USA).

The injected sample volume was 1 μL and the elution was performed at a flow of 0.3 mL/min using water with 0.5% formic acid added as solvent A and acetonitrile as solvent B. The elution gradient was varied as follows: at 0 min the solvent ratio was 90% of A and 10% of B, at 3 min 85% of A and 15% of B, at 6 min 75% of A and 25% of B, at 11 min 65% of A and 35% of B, at 16 min 50% of A and 50% of B, at 20 min 0% of A and 100% of B; this final composition was maintained for 8 min. Then, the system was brought back to the initial conditions and left to equilibrate with a post-time of 7 min. The total analysis time was 35 min, while the acquisition time was 28 min. The mass spectrum was acquired by ESI ionization in negative mode in the m/z range of 50–1600 with a scan rate of 1 spectrum/s, simultaneously infusing in addition to the eluent from the HPLC system (via the first nebulizer) the two reference masses (through the second nebulizer) having m/z 112.985587 and 980.016375. The parameters of the Dual ESI source were as follows: gas temperature, 325 °C; flow of the drying gas, 10 L/min; nebulizer pressure, 35 psig; capillary voltage, VCap 3500 V; Fragmenter, 150 V; skimmer, 65 V, octapole 1 RF, 750 V. The data were acquired in all-ion MS/MS mode by acquiring the chromatogram in full scan and in MS/MS with collision energy 10 V. For the analyses and data acquisition, Agilent MassHunter B software 06.00 was used. For the identification of compounds, several online libraries of MS and MS/MS spectra (METLIN, Human Metabolome Database or HMDB and mzCloud) were used.

### 2.5. Determination of Anthocyanins

PJ (5 mL) was acidified with 0.1% hydrochloric acid, filtered with CA 0.45-μm syringe filters (Whatman, Clifton, NJ, USA), and injected into HPLC instrumentation consisting of an Agilent Technologies Model 1100 system with quaternary pump, degasser, autosampler, thermostated column compartment, UV-Vis photodiode detector (DAD), and fluorescence detector (FLD). The ChemStation, also from Agilent Technologies, in addition to controlling the entire instrumentation, performed the processing of the chromatographic data. HPLC analysis of anthocyanins was carried out as reported by Mazza et al. [51]; the column used was an Inertsil ODS-3 150 × 4.6 mm with a particle diameter of 5 µm (GL Sciences Inc., Torrance, CA, USA); the injection volume was 20 µL. For the determination of anthocyanins, the DAD was set to a wavelength of 525 nm. The identification of anthocyanins was carried out on the basis of retention times and the characteristics of the UV-Vis absorption spectrum were as described in the literature [52]. The quantitative evaluation of the anthocyanins present in PJ was performed using cyanidin-3-O-glucoside as a standard.

### 2.6. Determination of Organic Acids

The samples were diluted to half strength with double-distil water filtered through CA 0.45-μm filters (Whatman, Clifton, NJ, USA) and injected into HPLC. The HPLC instrumentation was the same as that used for the anthocyanin determination. The chromatographic separation was carried out using a 250 × 4.60 mm Gemini (Phenomenex, Torrance, CA, USA) C18 column with a particle diameter of 5 μm, as reported by Kafkas et al. [53].

### 2.7. Evaluation of the Antioxidant Potential

The antioxidant power of PJ samples was evaluated as antiradical activity by three different assays. For each assay, the PJ samples were diluted 50-fold in water. The analyses were carried out using a Cary 100 Scan UV-Visible Spectrophotometer (Varian, Walnut Creek, CA, USA) and the results were expressed in μmol of TROLOX equivalent (TE) in mL of sample using the TROLOX calibration line (0.01–0.1 μmol).

#### 2.7.1. 2,2-Diphenyl-1-Picrilidrazil (DPPH^•^) Assay

A free radical test was carried out according to the method described by Brand-Williams et al. [54]. Briefly, 200 μL of sample were added to 3.8 mL of DPPH^•^ methanolic solution (25 mg/L). The absorbance of the reaction mixture (MR) was determined at 515 nm after 20 min at room temperature in the dark.

#### 2.7.2. Ferric Reducing-Antioxidant Power (FRAP)

The analysis was carried out according to Pellegrini et al. [55]. The methodology was based on the reduction of the Fe^3+^–TPTZ complex in ferrous form at low pH and = its monitoring by measuring the change in absorption at 593 nm. Briefly, 3 mL of FRAP reagent was mixed with 100 μL of sample. The absorbance at 593 nm was measured after an incubation of 30 min at 37 °C. The FRAP values were derived from the comparison between the absorbance variations in the test mixture with those obtained from increasing concentrations of Fe^3+^.

#### 2.7.3. 2,2′-Azinobis-(3-Ethylbenzothiazolin-6-Sulfonate) Acid (ABTS)

The test was carried out according to the basic method described by Re et al. [56], with the modifications in the dosages introduced by Ballus et al. [57].

The ABTS was dissolved in water up to a concentration of 7 mmol/L. The radical cation ABTS^•+^ was produced by reacting the initial ABTS solution with 2.45 mmol/L of potassium persulfate solution at a 1:1 volume ratio and subsequently incubating this mixture in the dark at room temperature for 12–16 h before use. The working solution was prepared by diluting the solution described above with HPLC-grade ethanol until an absorbance of 0.700 ± 0.020 at 734 nm was reached, and then balanced at 30 °C. Briefly, 30 µL of extract was placed in a tube together with 3 mL of ABTS^•+^ solution (A734 nm = 0.700 ± 0.020). The absorbance was read at 734 nm after 6 min. The % inhibition of absorbance at 734 nm was calculated and plotted as a function of the concentration of antioxidants and TROLOX for standard reference data.

### 2.8. Evaluation of Volatile Compounds

The composition in volatile substances of the PJ head space was determined by a mass spectrometry (MS) analysis coupled with gas chromatography (GC) through the headspace by means of solid-phase microextraction (SPME). The PJ (2 mL) was placed in a 20-mL vial with 25 μL of internal standard (4-methyl-2-pentanol 750 µg/L), hermetically sealed, and placed in the autosampler. SPME sampling of volatile compounds was performed by exposing the fibre consisting of Carboxen/divinylbenzene/polydimethylsiloxane 50/30 μm × 2 cm long (Supelco Inc., Bellefonte, PA, USA) for 30 min in the sample’s headspace at 25 °C. Before adsorption, the sample was stirred (750 rpm) for 30 min at 40 °C. The adsorbed compounds were then thermally desorbed for 5 min by inserting the fibre into a GC injector heated at 250 °C. The analyses were conducted with an Agilent Technologies 7890B GC equipped with a 7693A Multimode Injector (MMI) and a PAL3 RSI 120 thermostated autosampler equipped with a fibre conditioning module and agitator (CTC Analytics AG, Zwingen, Switzerland) coupled with a single MS 5977B quadrupole (MSD) with XTR (Extractor Ion Source) electronic impact source (Agilent Technologies, Palo Alto, CA, USA). For the separation of volatile compounds, a capillary column in fused silica DB-WAXetr with a length of 50 m was used, with an i.d. of 0.32 mm and a film thickness of 1 μm (Agilent Technologies). Helium was used as carrier gas at a flow of 1.7 mL/min, which was kept constant throughout the analysis by an electronic flow control device (EFC). The GC column oven temperature was set according to the following schedule: Initial temperature 35 °C held for 4 min, then increased to 45 °C at a rate of 5 °C/min, further increased up to 150 °C at a speed of 4 °C/min, increased again up to 180 °C at a speed of 8 °C/min and maintained for 2 min and finally brought to 210 °C at a speed of 11 °C/min and maintained for 13 min; under these conditions, the total analysis time was 55 min. The injector was set to a temperature of 250 °C. The transfer line temperature was set to 215 °C; as regards the experimental conditions of the mass spectrometer, the temperature of the source was 190 °C and that of the quadrupole was 150 °C. The electron impact mass spectrum (EI) was recorded with an ionization energy of 70 eV in the mass range 25–350 a.m.u., 4.3 scan/s; the MS spectra were acquired in scan mode. The processing of the collected data was carried out using Agilent MassHunter B.08.00 software with the Unknown Analysis module. The identification of volatile compounds was carried out by comparing the mass spectra and retention times thus obtained with those of pure analytical standards and with the spectra of the NIST-2014 library. The volatile compounds were quantified, and expressed in µg/kg, by comparing the area of the extracted ion of each peak with the area of the peak of the internal standard ion (4-methyl-2-pentanol), as reported by Xiao et al. [58].

### 2.9. Sensory Analysis

PJs were subjected to a descriptive–quantitative sensory analysis by a panel of 10 people previously trained in sensory analysis according to the ISO 8586: 2012 standard [59] and in the descriptive sensory evaluation of fruit juices and of pomegranate-based products. For the present study, the panel worked during two orientation sessions (90 min each), discussing the main sensory characteristics of commercial juices and pomegranate products. The lexicon used to describe positive attributes and off-flavours was based on that previously developed by other authors [18,60,61,62] and subsequently re-elaborated during the orientation sessions. The 37 selected attributes (Table S3) were divided into four groups, namely appearance, smell, texture/mouthfeel, and taste. All sessions were carried out in a sensory room at 20 °C under normal (warm or cold) lighting conditions. The samples were served in 90-mL covered odourless plastic cups at room temperature (Figure S1). Assessors received and tested the samples in a balanced order, reporting their quantitative evaluations on an unstructured scale, with a 9-cm-long line for each descriptor. The results were included in the multivariate statistical analyses (PCA and PLS models).

### 2.10. Statistical Analysis

The data obtained from the analysis of PJs were statistically processed using univariate and multivariate statistical approaches.

The statistical analysis of the variance of the data was carried out through one-way ANOVA using the Tukey test (*p* < 0.001). The software used was SigmaPlot v. 12.3 (Systat Software Inc., San Jose, CA, USA).

The multivariate statistical analysis was carried out on the entire dataset using the principal component analysis (PCA) and the partial least squares projections to latent structures (PLS).

For the multivariate modelling, data were first normalized by autoscaling in order to give all the variables the same weight. Furthermore, to obtain a distribution that was as close as possible to the Gaussian one, some of the variables were previously transformed logarithmically using the logarithm function in base 10. Cross-validation was used to establish the number of significant components in the definition of the model.

All multivariate statistical processes were carried out using the chemometric package SIMCA v. 13.0.3.0 (Umetrics AB, Umeå, Sweden).

## 3. Results and Discussion

### 3.1. Total Phenolic Compounds and Tannins

According to the total phenolic compounds results (Table 2), significant differences (*p* < 0.001) existed between from concentrate and not from concentrate juices, with ranges of 1379–3748 mg GAE/L and 1632–2736 mg GAE/L, respectively, reflecting the results found by many authors in which a higher concentration of total phenols was always recorded in juices that had undergone some type of concentration process [4,5,18,21]. However, those levels of polyphenols were always lower than those revealed by some authors [19,20,24] on not from concentrate and not thermally stabilized PJs. Furthermore, our results seem to indicate a larger variability in the juices from concentrate than in the not from concentrate ones.

A broad variability in the phenolic profile and content of these compounds was reported in the literature [3,4,5,6,7,8,9,10,11,12,13,14,15,16,17]. For instance, on the one hand, Nuncio-Jáuregui et al. [18] found a narrow range of total phenolic concentration (2285 to 2457 mg GAE/L) among fresh and commercial typical Spanish PJs (obtained from conventional and organic agricultural practices); on the other hand, Mena et al. [5], analysing 15 fresh PJs, observed considerable variation among typical Spanish cultivars (CVs) and those from around the world, with more diffused ‘Wonderful’ CV (1562–4500 mg GAE/L). Moreover, the range of total phenolic compound concentrations evaluated in seven commercial PJs from Turkey (ranging from 2602 to 10,086 mg GAE/L) were larger when compared to the data shown in our work [63]. The wide variability of total phenolic compounds found in PJs could be partially ascribed to agronomic and technological factors [3]. In this regard, Labbé et al. [20] evaluated several PJs obtained from three different varieties (‘Wonderful’, ‘Chaca’ and ‘Codpa’), harvested at different maturity stages (unripe, medium ripe, and full ripe), in terms of chemical composition, total phenolic content, antioxidant capability, and anthocyanin profile [20]. They observed that the PJs’ total phenolic content did not vary significantly with the maturity stage but was strongly affected by the cultivar [20]. Similar results were found by Tehranifar et al. [22], pointing out the importance of the cultivar, which affects the physiochemical properties and antioxidant activity in pomegranate juice.

Likewise, the impact of the PJ extraction process on the phenol composition has been intensively investigated [26,64]. Indeed, Gözlekci et al. [19] reported on the total phenolic distribution of juice, peel, and seed extracts of four popular Turkish pomegranate cultivars (‘Lefan’, ‘Katirbasi’, ’Cekirdeksiz-IV’, and ‘Asinar’). The PJ obtained from the whole fruit, including the rind or husk, showed higher total phenolic content (2566 mg/L) than that produced from arils only (1800−2100 mg/L). These findings suggested a transfer of these compounds from the pomegranate membrane and rind to the juice during whole fruit pressing [19,21].

Among phenolic compounds, the percentage ratio occupied by tannins was almost always higher in from concentrate juices (average: 32.6%) than in ones not from concentrate (average: 25.9%). The greater presence of tannins in CJ samples may be caused by the same concentration process or by the extracting method involving the whole fruit [5,21]. Comparing values according to the cultivation method, no significant differences were found among organic and conventional juices, neither for total phenols nor the tannins fraction. Our results are in agreement with those of Nuncio-Jáuregui et al. [18], who pointed out that total phenolic compounds are not significantly affected by the farming type. On the contrary, Cano-Lamadrid et al. [65] observed a higher total phenolic content for PJ extracted using organic growing methods when compared to conventionally grown pomegranate fruits. The differences found for the total phenolic compounds content could be partially due to the combined effects of different conditions, such as accessions, cultivar, environmental conditions, and juice processing. However, further studies are needed to clarify the effect of organic or conventional growing conditions on the total phenolic content.

### 3.2. Ellagitannins and Precursors

Compared to other fruits, the pomegranate is richer in phenolic compounds, mainly hydrolyzable ellagitannins (punicalins and pulicalagins, in particular), which are responsible for its health-promoting and sensory properties [7,8].

Table 3 shows the values of hydrolyzable tannins (in particular, ellagitannins) and their derivatives, such as ellagic acid, in the eight commercial juices. Among all these molecules, the most abundant in all the samples were punicalins and punicalagins (in particular, the former), which constitute 71–85% of the total content of this phenolic fraction, except for INCPJ2 (30%, with a general lower phenolic content).

The sum of punicalins a and b, between 712 mg/L and 1841 mg/L (excluding the INCPJ2 sample), was higher than the values found in the literature on nonpackaged fresh PJ [66]. Valero et al. [66] reported that punicalins content in PJs clarified by microfiltration (MF) and ultrafiltration (UF) technologies compared to conventional method was 710 mg/L, 663 mg/L, and 703 mg/L, respectively [66]. Concentrations of punicalagins, between 21 mg/L and 177 mg/L (excluding the INCPJ2 sample), were, on the contrary, in line with those found by other authors [4,18]. In particular, Nuncio-Jáuregui et al. [18] reported that the punicalagins levels of commercial PJs from ‘Mollar de Elche’ CV grown under conventional or organic farming ranged from 100 to 200 mg/L. Moreover, while the ellagitannins fraction fluctuated from 1047 to 1625 mg/L and from 596 to 2374 mg/L in NCJ and CJ, respectively, punicalins and punicalagins values differed significantly. Furthermore, they were higher in CPJs, probably because of the water reduction effect. No differences were observed when comparing the PJs according to the pomegranate fruits’ cultivation (conventional or organic methods).

The ellagic acid content was found to be in the range of 22.1–69.8 mg/L, representing 3–4% of the tannic phenols. These findings are in agreement with those of Todaro et al. [16], who observed a wide variability in ellagic acid content, typically between 16.1 and 104.3 mg/L.

With respect to the punicalagin content, Gil et al. [4] estimated that it was generally higher in commercial juices (measuring up to 2 g/L) than in fresh ones, and that this was due to the processing method; thus, the high content in commercial juices could be attributed not only to the transfer of punicalagins from the husk, but also to the thermal treatment [4]. Fisher et al. [21] also observed that the punicalagins content increased due to the higher pressures applied to the fruit during whole fruit extraction. In particular, their concentration was at least 10.5-fold higher in juice prepared from whole pomegranates at 150 bars compared to those at 10 bars. Moreover, Mena et al. (2012) [34], comparing two different thermal treatments (high-temperature, short-time (90 °C, 5 s) and low-temperature, long-time (65 °C, 30 s)) in PJs from the ‘Mollar de Elche’ and ‘Wonderful’ CVs., reported a significant increase in the punicalagins content in all pasteurized juices with respect to fresh ones, while the ellagic acid content decreased by ~60%. They suggested that the punicalagins formation could be correlated to the degradation or breakdown of larger-molecular-weight compounds, whereas the thermal treatment negatively affected the content of ellagic acid [64].

### 3.3. Anthocyanins

Anthocyanins belong to the flavonoids class and are the main pigments found in the plant kingdom. Their colours vary from pink through red, purple, and blue, depending on the pH. Besides their use as natural food colourants, the potential health benefits of anthocyanins have been widely demonstrated in the prevention of cardiovascular, neurological, and other chronic diseases, exploiting their antioxidant and anti-inflammatory activities [8,19]. However, anthocyanins are characterized by a strong reactivity, and therefore undergo degradation reactions, mainly due to oxygen, light, temperature, enzymes, and storage time [17]. The anthocyanins determined during the analysis were delphinidin and cyanidin, both in the 3-glucuside and 3,5-diglucoside forms, except for INCPJ2 (Table 4). Unexpectedly, in the INCPJ2 sample (HPP-processed and packaged in a 200-mL PET bottle), anthocyanins were not detected. It could be supposed that HPP treatment did not induce total inactivation of the enzymes involved in the anthocyanin’s degradation. Thus, their residual activity was triggered by a low concentration of dissolved oxygen (due to the gas permeability of PET), causing the total degradation of these molecules. In fact, this loss was not observed in the HPP-treated sample that was stored in glass (INCPJ4).

**Figure 1:**
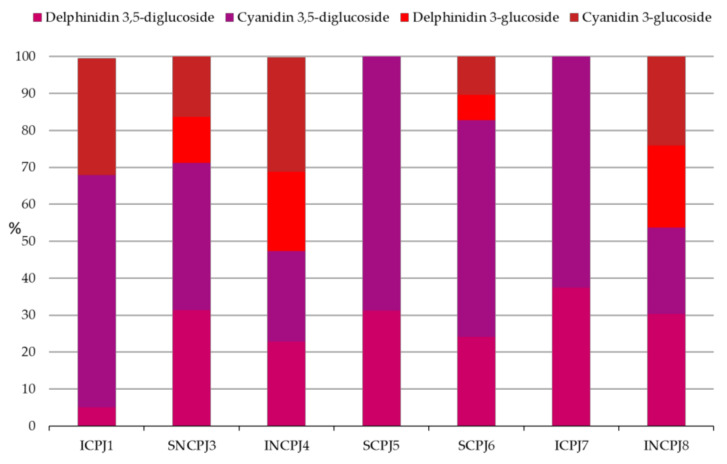
Distribution (%) of the anthocyanins of PJs analysed.

The most abundant anthocyanin was cyanidin, in particular in its 3,5-diglucoside form, in agreement with the findings of other authors [6,20]; specifically, in CJ samples, it represented more than 60% of the total anthocyanins, while in the NCJ samples, the ratio between the various anthocyanins was more balanced (Figure 1).

However, the total content of anthocyanins in CJs was much lower (0.7–47 mg/L), in particular in SCPJ5, SCPJ6, and ICPJ7, than that in NCJs (59.7–280.6 mg/L), with the most abundant forms represented by 3,5-diglucoside. Among all samples, the highest number of anthocyanins was revealed in INCPJ8, belonging to the NCJ group. It is feasible to assume that the lower anthocyanin concentration in CJ samples is due to the concentration process, which, involving higher temperatures, causes their loss because of their oxidation and transformation into quinones or their degradation, remaining in low quantities in the final juice. These results confirmed previous data obtained by Fischer et al. [67], who investigated the thermal stability of anthocyanins in three PJs by heating them to 60 °C, 70 °C, 80 °C, or 90 °C, for 15 min to 5 h. They found a significant decrease in the anthocyanin content during heat treatment between 60 °C and 90 °C. The same authors also estimated that the thermal degradation of the anthocyanins from the PJ was characterized by a first-order kinetic. The findings described above were also corroborated by the fact that the ICPJ 1 sample, which was treated with a mild concentration (under high vacuum water evaporation), showed higher levels of anthocyanins, confirming what was observed by Yousefi et al. [40] and Dhumal et al. [39]. These authors compared the effect of different concentration methods (including conventional and microwave heating [39,40] and the rotary vacuum evaporation technique [39]) on the evaporation rate and quality attributes of PJ. In both studies, the degradation rate of anthocyanins, colour, and antioxidant activity was found to be faster when using a conventional heating method than a non-thermal one [39,40].

### 3.4. Antioxidant Activity

The values of antioxidant activity determined by DPPH^•^, FRAP, and ABST found in the literature were often interchangeable, even if they are dependent on various factors related to the juice chemical composition as well as the methodology used. Indeed, our research demonstrated that DPPH^•^, FRAP, and ABTS values were similar in each sample analysed (after normalizing the data) (Table 5).

Furthermore, the three methods confirmed that the PJ antioxidant activity is strongly correlated with the phenolic content [4,5,6,8,24,68], demonstrating high R values (0.96, and 0.97 for DPPH^•^ and FRAP, respectively (*p* < 0.01)) and lower ones for the ABTS assay (0.70) (*p* > 0.05)), with no statistical differences between data on the two types of juices (NCJ and CJ samples), as observed for the total phenolic composition (Table 2). However, no statistical correlation was found between anthocyanins content and antioxidant activity; this means that the antioxidant activity is mostly independent of anthocyanins but exhibited by other molecules such as hydrolyzable tannins due to the number and position of OH groups in their molecular structures. This agrees with the findings of Tzulker et al. (2007) [68].

In fact, hydrolyzable tannins showed a high correlation (R = 0.95, 0.96 and 0.76 (*p* < 0.01)) with the antioxidant activity measured by DPPH^•^, FRAP, and ABTS.

By comparing our results with those in the literature regarding unpackaged fresh PJ, similar and often higher values were observed [6,68,69].

In particular, the antioxidant activity data obtained by Wern et al. [69] for fresh and commercial PJs, via the DPPH^•^ and FRAP methods (267.78 and 292.13 μmol TE/100 mL of juice, respectively for fresh juices, and 2705.01 and 2953.85 μmol TE/100 mL of juice, respectively, for commercial ones) confirmed, in part, our results.

On the other hand, the results of Di Stefano et al. [6] revealed that the total antioxidant activity values, evaluated by ABTS, lay between 36.73 ± 4.1 and 221.5 ± 10.0 μmol TE/100 mL juice, in line with our data obtained via the same assay.

They also found a high positive correlation between the total antioxidant activity of PJs of five genotypes (‘Mollar’, ‘Kingdom’, ’Dente di Cavallo’, ’Francofonte’, and ’Santa Tecla’), evaluated using the ABTS assay and the total phenolic compounds, pointing out that a significant number of phenolic compounds represents a relevant contribution to the antioxidant capacity of these PJs.

### 3.5. Organic Acids

It is well known that organic acids (malic and citric acid, in particular) play an important role in preserving food and beverage quality, enhancing shelf life by inhibiting the growth of food spoilage and pathogenic microorganisms, and improving the sensory (flavour, colour, and aroma) as well as health properties (like antioxidants and antimicrobial activity) [70].

Malic and citric acids were evaluated due to their higher concentrations in the organic acids fraction of the PJs [71] (Table 6). The results demonstrated the high quantity of citric acid in all the samples analysed, with values ranging between 4.7 g/L and 14.7 g/L. Furthermore, both the sum of the two acids and the quantity of the single ones were in line with data found by several authors for fresh, unpackaged PJ from different cultivars of fruits [71,72]. Moreover, besides sample 7 (which showed the highest content of acids, 19.3 ± 0.2), significant differences were found between NCJ and CJ samples in terms of the total organic acid content (Table 6), with higher values for the former—an average of 12.55 ± 1.40 g/L. These data could be explained by the sensitivity to degradation that these compounds show toward the traditional concentration process [71]. This sensitivity was also seen at a lower temperature with water evaporation during concentration since similar values were found for ICPJ1 (treated under a vacuum system) with respect to other CJs.

### 3.6. Volatile Compounds

Pomegranate fruits have been recognized as having low aromatic intensity. The literature data show that, although the chemical classes of the main aroma compounds are similar (alcohols, aldehydes, terpenes, esters, furans, and acids), the abundance of specific compounds varies significantly according to the variety, growing area, ripening stage, and processing conditions [28,32,33]. Specifically, a mixture of different volatile compounds, including alcohols (hexanol and (Z)-3-hexenol), aldehydes (hexanal), and terpenes (β-pinene, limonene, α-terpineol, and β-caryophyllene), plays an important role in contributing to the pomegranate fruit flavour. They provide ‘green’, ‘woody’, ‘earthy’, ‘fruity’, ‘floral’, ‘sweet’, and ‘musty’ notes [73].

Moreover, the volatile compounds lost during juice manufacturing lend to the commercial PJs’ different characteristics with respect to the fresh ones [73]. The heat treatment adopted either for the juice concentration process or for the microbial stabilization leads to a reduction in ester levels; a slight increase in terpenes compounds, which contribute to the fresh flavour; and an increase in furans compounds such as 2-furfural and 5-methyl-2-furfural, resulting from Maillard reactions [29]. Finally, some minor compounds (such as 3-methylbutanal, 1-pentanol, (Z)-3-hexenyl acetate, methyl benzoate, and eucalyptol), thanks to their low odour threshold, contribute in a non-negligible way to the PJ aroma, as can be seen in Table 7.

For this reason, more recently, the aroma profile of commercial PJs was investigated [24,28,32,73,74,75].

In our research, a SPME-GC/MS analysis of the PJs’ head spaces allowed us to find 27 volatile compounds represented by five aldehydes, seven alcohols, one ketone, two esters, three acids, four furans, and five terpenes (Table 8). From a quantitative point of view, NCPJ samples showed higher total numbers of volatile compounds, with a concentration ranging between 1272 µg/L and 4002 µg/L, versus a range of 286.3 µg/L and 1020 µg/L found in the CPJ samples. This finding could be justified by supposing that the volatiles lost during the concentration process were recovered and added back to the juices at the end of the concentration process.

**Figure 2:**
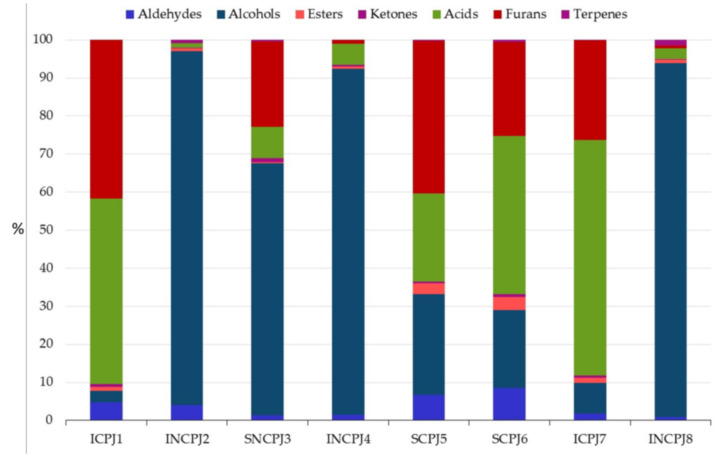
Distribution (%) of the volatile compounds of PJs analysed.

On the other hand, when observing the single classes of compounds (Figure 2), alcohols were the main classes characterizing the volatile profile of NCPJs, representing 86% of the total content; on the contrary, in CPJs we find 44% of acids (in particular, acetic acid) and 33% of furans (mainly 3-furfural), which were found in traces in NCPJ samples, except for SNCPJ3. Regarding the alcohol fraction of NCPJs, these were represented almost exclusively by ethanol (96% of the total), which usually characterizes alcoholic beverages; it is feasible to assume some anomalous fermentation in the fresh PJs before the successive treatments. Hexanol and 3-hexen-1-ol, found at high concentrations by many authors in fresh, unpackaged PJs [74], were also discovered at much higher concentrations in NCPJs than in CPJs, probably because of their degradation due to the heating process undergone during the concentration phase.

The other families of volatile compounds constituted a small part of the total content: aldehydes represented 3.7%, with benzaldehyde and 3-methylbutanal as the most abundant, except for sample 3, where higher concentrations of acetaldehyde were found.

Esters, almost exclusively represented by methyl benzoate, contributed 1.4% of the total.

The only ketone found was acetoin, with 0.5% of the total volatile compounds content. We found a large number of terpene components in all the juices analysed, except for ICPJ1, confirming previous results [73], even if this fraction constituted only 0.5% of the total volatile compounds content; among them, we mainly found α-terpineol and one of its isomers, 4-terpineol, also discovered by other authors [75].

In this regard, terpenes represent important fingerprint compounds correlated with the particular variety of fruit [32]. Furthermore, even if their presence was limited to a lower concentration than the other volatile classes, they are usually characterized by a very low sensory perception threshold; thus, they are very important for providing the typical sensory aroma of products such as juices. The fact that we did not find any traces of these terpenes in ICPJ1, which, in addition, was from an unknown origin, led us to suppose the particular fruit variety was not characterized by these compounds.

Regarding furan substances, their presence in products like PJs is due to the Maillard reaction arising during heat treatments (concentration or microbial stabilization) for the degradation of carbohydrates such as glucose or fructose, amino acids, polyunsaturated fatty acids, and organic acids [89]. In particular, Kus et al. [90], when analysing PJ subjected to high temperatures, found furan compounds up to 3500 ppm; this is caused by the low pH of the medium, which facilitates the formation of these molecules when it is heated.

In our research, we found furan compounds in all samples (except for INCPJ2), even if at low percentages of the total content (3.4%), except for SNCPJ3, where these molecules make up 22% of the total volatile compounds. The high concentration in furans in SNCPJ3 may have been caused by too lengthy a pasteurization process.

On the contrary, the absence of furans in the INCPJ2 head space was correlated to the mild process used for stabilizing the juice product. This finding led us to emphasize the suitability of this mild technology for preserving the original aroma.

### 3.7. Sensory Analysis

A sensory analysis of the eight commercial pomegranate juices was carried out by the QDA method. Data collected were firstly graphically elaborated, comparing all the juices in four different spider plots for appearance, smell, taste, and tactile sensory attributes, respectively. As reported in Figure S2, a clear distinction was revealed by the assessors between from concentrate and not from concentrate juices. For those attributes concerning the appearance (see Figure S2a), NCPJs obtained high values for ‘red–purple’ colour (median: 4.3–6.8) and ‘turbidity’ (4.9–5.9) and lower levels for the ‘red’ colour attribute (2.05–3.3); opposite data were observed in CPJs, which had values of 5.2–7.2, 0.7–3.0, and 1.2–4.8, respectively, for the attributes ‘brick–red’ or ‘purplish–red’ colour and ‘turbidity’ (see Figure S2a). These results can be explained by considering that the concentration process, carried out at high temperatures, even if operating in a partial depression, leads to the degradation and oxidation of anthocyanins (the molecules most responsible for the PJ colour) and the consequent formation of quinones with a characteristic brown appearance. Furthermore, the lower level of the ‘cloudy’ attribute in CPJs can be explained by the fact that concentration processes are often preceded by filtrations and clarifications, with the aim of improving the concentration process, and these can also be repeated in the subsequent juice dilution phase before the pre-packaging heat treatment.

Analysing the results obtained by the olfactory sensory analysis (see Figure S2b,), we observed that the overall aromatic intensity of the juices was similar for both types but NCPJs were mostly characterized by the following sensations and relative values: 2.9–5.2 for ‘pomegranate,’ 2–3.5, for ‘fresh fruit,’ and 2–4.5 for ‘beetroot’ attributes, which can be related to the typical sensation of the fresh fruit [18,61,73]; on the contrary, the most representative olfactory attributes found in the CPJs were ‘dried fruit’ (2.8–5.5), ‘candies’ (1.7–4.3), ‘caramel’ (2.4–3.6), ‘honey’ (2.8–5), and ‘cooked’ (3.8–5.5), all of these potentially related to the Maillard reaction during the concentration process, which is usually responsible for the production of furans and the ‘caramel,’ ‘honey,’ and ‘cooked’ aromas [61].

Moreover, in the CPJs, ‘red fruity’ aromas such as ‘cherry’, ‘plum’, or ‘berry’ did not have high values, remaining in all cases under the value of 3.

As regards the attributes related to the texture/mouthfeel, differences were observed in the form of more homogeneity (4.7–5.6) for NCPJs and a greater ‘overall intensity’ (with values of 4.8–5.5 compared to 2.6–4) for CPJs, demonstrating that the high heat treatment and subsequent dilution not only alter the colour but also the flavour of the juice (see Figure S2c,). Few works can be found in the literature regarding the sensory analysis of PJ; these are almost exclusively comparing freshly squeezed juice with those that can be found on the market, not specifying the treatment they have undergone. Koppel and Chambers [61] found high values in the ‘brick–red’ attribute and ‘candy’ aroma in from concentrate juices, whereas in not from concentrate juices there were higher values of the ‘berry’ aroma and the ‘deep red’ to ‘purplish’ colour [61].

Comparing fresh and packaged juices, Nuncio-Jáuregui et al. [24] observed a much higher aromatic intensity, with higher values of ‘fresh fruit’ or ‘pomegranate peel’ attributes in the former and a marked aroma of ‘cooked’ and ‘mushroom’ in the latter. Fresh juices presented stronger astringency. This led us to suppose that, in general, storage stabilization techniques (even mild ones) lead to a change in the juice’s sensory profile, with a loss of the typical pomegranate fruit colour and the appearance of sensory characters such as ‘brown’ colour and ‘cooked’ flavour and a general loss of typical aromas of fresh PJ, such as ‘berry’, ‘red fruit’, and ‘green’.

### 3.8. Multivariate Analyses

In order to understand how the various instrumental and sensory measurements carried helped us to discriminate between the analysed samples and to observe any correlations between chemical and sensory components, two different multivariate statistical analyses were carried out, i.e., the principal component analysis (PCA) and the partial least of square of regression (PLS) analysis.

Regarding the PCA, the refined model was built with eight samples and 78 variables, explaining 64% of the total variance in the data (with two principal components, P1 and P2, accounting for 39% and 25% of the variance, respectively).

**Figure 3:**
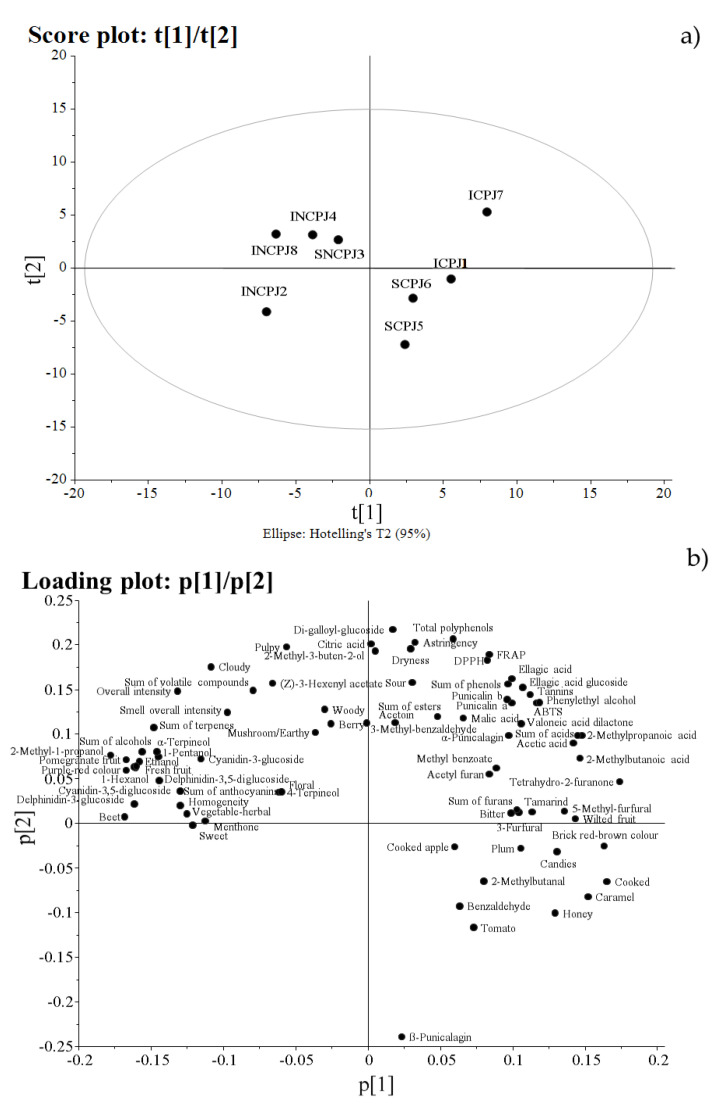
(**a**) Score and (**b**) loading plots of principal component analysis (PCA) model.

From the score plot relating to these components (Figure 3a), the most important separation among the objects (juice samples) was relative to the technological process of concentration. In fact, as observed along the first component (from the left to the right side of the score), samples were separated into two distinct clusters. NCPJs were located on the left side of the score and CPJs were located on the right side (Figure 3a). The other four samples, corresponding to the organic origin (not EU) CPJs were located on the opposite side (the right side) of the score (Figure 3a).

From the relative loading plot (Figure 3b), we observed that the variables with the highest loadings, and hence the most responsible for the left location of NCPJs in the score plot, were: 2-methyl-1-propanol, delphinidin-3,5-diglucoside, delphinidin-3-glucoside, the sum of anthocyanins, alcohols (including hexanol and their sum), α-terpineol, menthone, and the sum of terpenes; and sensory descriptors ‘beetroot’, ‘pomegranate fruit’, ‘purplish–red colour’, ‘fresh fruit’, and ‘overall intensity’. On the contrary, the variables characterized by the highest loading, responsible for the right-hand location on the score plot of the CPJs, were: tetrahydro-2-furanone and the sum of acids as well as sensory attributes like ‘cooked’, ‘brick red/brown colour’, ‘caramel’, ‘dried fruit,’ ‘candies’, and ‘honey’ (Figure 3b). Thus, the PCA allowed us to confirm that the technological phase of concentration affected the chemical composition and sensory characteristics of the juices more than other factors such as the fruit origin, cultivation method, or distribution system. Furthermore, PCA allowed us to focus on those variables most responsible for the juice’s differentiation, confirming that the parameters usually correlated with the juice freshness [24] were the same as those characterizing our NCPJs. On the contrary, CPJs, besides having lower levels of the freshness parameters, were also characterized by the typical sensory aspects arising from the juice concentration phase [24].

Moreover, a PLS model was built for investigating the influence of processing on the PJs’ quality, and in particular on the correlation between instrumental and sensory data, which we previously illustrated separately.

**Figure 4:**
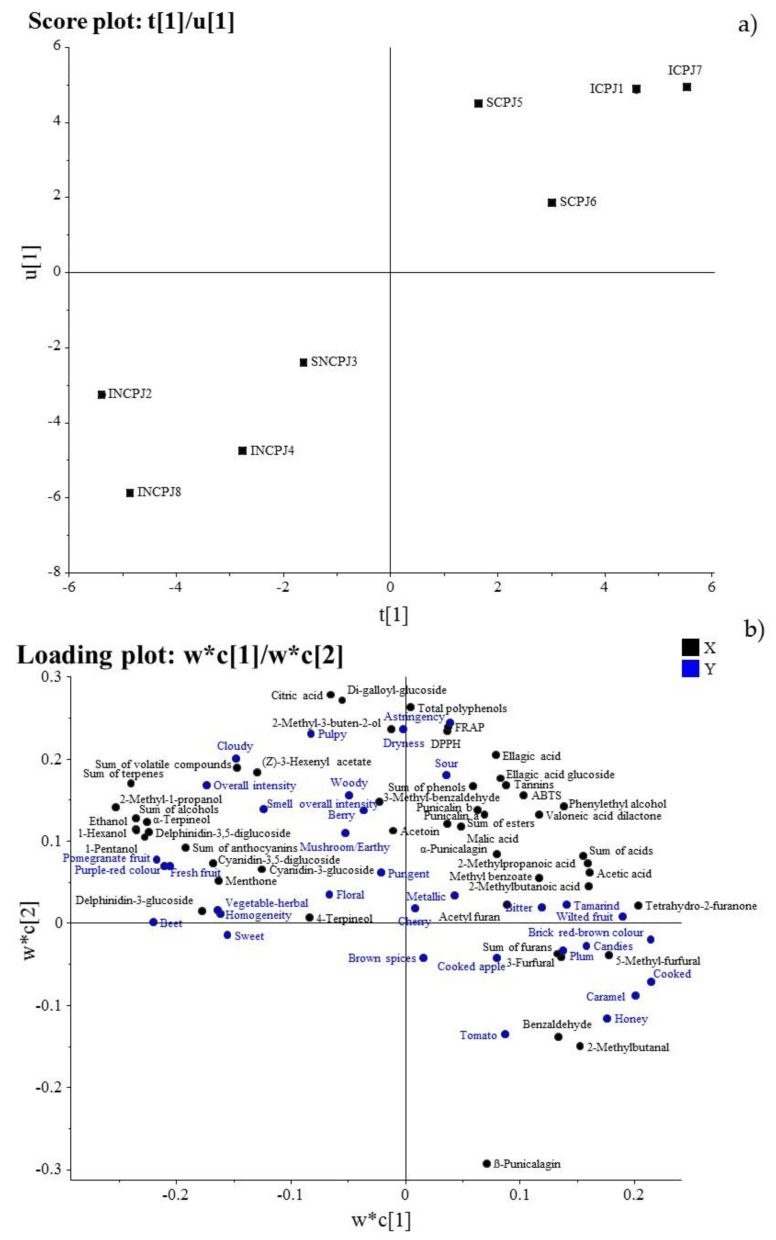
(**a**) Scores plot (t1/u1) of the first latent variable and (**b**) loading plot w*c[1]/w*c[2] of the partial least squares (PLS) model.

The PLS model obtained after normalizing and autoscaling the original data (Figure 4) explains 81% of the total variance of the Y variables (with four components.) From the score plot of the first component U of block Y and the first component T of block X (Figure 4a), we can see a clear distinction of the objects, firstly according to the treatment and secondly to the fruit origin, observing a trend that goes from samples INCPJ2 and INCPJ8 and, to a lesser extent, INCPJ4, placed in the lower left corner; on the opposite corner are samples ICPJ7, ICPJ1, and SCPJ5, representing the concentrated juices. No distinction of the samples was revealed according to the fruit cultivation method and the juice distribution.

From the relative loading plot of the first two components (Figure 4b), high positive correlations were observed between the chemical parameters 2-methyl-1-propanol, sum of terpenes, sum of alcohols, ethanol, 1-hexanol, 1-pentanol, α-terpineol, delphinidin-3,5-diglucoside, sum of anthocyanins, delphinidin-3-glucoside, cyanidin-3,5-diglucoside, menthone and the sensory attributes ‘beet’, ‘pomegranate fruit’, ‘purple–red colour’, ‘fresh fruit’, ‘overall intensity’, ‘homogeneity’, and ‘vegetable herbal’ characterized by the maximum values for the NCPJs, which represent the first PLS latent variable (the left side of the score plot, Figure 4b).

On the opposite side, high positive correlations were observed between the chemical parameters tetrahydro-2-furanone, 5-methyl-furfural, acetic acid, 2-methylbutanoic acid, sum of acids, 3-furfural, 3-methylbutanal, benzaldehyde sum of furans and the sensory attributes ‘cooked’, ‘brick red/brown colour’, ‘caramel’, ‘wilted fruit’, ‘honey’, and ‘candies’, for the second PLS latent variable represented by the CPJs, which showed the highest values for both of those parameters and the lowest values for those characterizing the NCPJs.

When a dependent variable is specified (sensory attributes in our case), the PLS technique has often demonstrated better performance than PCA due to the supervised nature of its algorithm. This provided insight into the separations between the CPJs and NCPJs. The chemical and sensory parameters contributing to the differences were anthocyanins, which correlated with the typical colour of the fresh fruit, and terpenes and alcohols, which showed close correlations with the typical odour of the fresh fruit. Furans (mainly furfural) have been related with Maillard reactions and the typically unpleasant odour of processed foods.

Our results seem to confirm that some compounds (aldehydes and furans, in particular) developed during the concentration process and the loss of some others (terpenes, alcohols, and anthocyanins) could mask some attributes expected in the PJs (i.e., ‘purple–red colour’, ‘beet’, ‘fresh fruit’, ‘pomegranate fruit’, etc.), which, on the contrary, are probably correlated to the concentration of 3-methyl-benzaldehyde, 1-pentanol, 1-hexanol, 3-hexen-1-ol, 4-terpineol, α-terpineol, and menthone. Similar conclusions were reached by Vázquez-Araújo et al. [73], who evaluated the volatile and sensory profile of 13 commercial PJs compared with freshly squeezed juice. They also investigated the relationships between these two analyses using a PLS. In particular, they observed that the freshly squeezed juice was mainly characterized by high values of terpenes, esters, and aldehydes, which seemed to be correlated with ‘fruity–dark’, ‘floral’ and ‘fruity’ notes. While furans were important contributors to the commercial PJs’ aromas, which were correlated with negative sensory attributes (i.e., ‘molasses’ and ‘candy-like’) [73].

## 4. Conclusions

PJ has been experiencing ever-increasing demand in recent years thanks to the numerous bioactive substances contained within it and exponentially growing consumer interest in functional and super foods.

However, due to the limited availability of fresh fruit at certain times of year and the difficulty of extracting juice at home, the consumer often falls back on the purchase of juices that are frequently produced starting from concentrated PJs.

To the best of our knowledge, this research has allowed, for the first time, for a comparison of the overall qualities of different types of pomegranate juices currently available at a supermarket or online.

According to our results, the most important factor that determines differences in health and sensory properties is the technological process of the concentration.

In fact, we concluded that, even if it was carried out in mild conditions (under vacuum water elimination), it promoted an important modification of the chemical composition and, thus, of the health properties and sensory characteristics of the final product. Indeed, we supposed that the sensitivity to high temperatures of some fractions of pomegranate phenolic compounds such as anthocyanins and several families of volatiles led to their reduction in the juice concentrate and, as a consequence, in the relative final product after reconstitution. The higher levels of these compounds in the NCPJs confirmed our hypothesis.

However, we also revealed that the concentration processes did not affect the phenolic fraction represented by hydrolyzable tannins, which, on the contrary, were often significantly (*p* < 0.001) higher than those found in NCPJs. This showed that 100% pomegranate juices from concentrate can also provide health benefits for consumers to correlate with the punicalins and punicalagins content, which usually represent the most important part of the hydrolyzable tannins. In this regard, even in CPJs, the hydrolyzable tannins were principally characterized by those forms.

Furthermore, through PLS models we could observe positive correlations between chemical and sensory parameters such as between anthocyanins and the typical colour of the fresh fruit and between terpenes and alcohols and the typical odour of the fresh fruit. On the other hand, we also observed positive correlations between negative sensory attributes (such as ‘brown’ colour and ‘caramel’ and ‘candy’ smells) and Maillard reaction products such as furfural compounds as well as benzaldehyde.

Finally, this research offers important indications to both consumers and producers: for the former, we can confirm that some aspects of the sensory attributes of pomegranate juice can be correlated to the quality of commercial pomegranate juices. Indeed, the colour, for example, can be used as a good indicator of the overall quality of the product.

For the latter, our results indicate that, besides the higher resistance of hydrolyzable tannins to the concentration made up at high temperatures, many other substances, such as anthocyanins and several volatile classes such as terpenes, alcohols, and aldehydes, are strongly reduced by these treatments, and are also responsible for the negative Maillard reaction. Thus, the possibility of introducing mild techniques involving cold concentrations such as micro- or ultrafiltration and cryoconcentration, might be an interesting strategy for improving the quality of the concentrated juice. To confirm this last hypothesis, analyses should be done on concentrated juices by cold concentration methods.

In this regard, producers who already apply or will apply these cold, mild methods of concentration should report this information on the label in order to inform the consumer of the attention paid to obtaining a higher-quality product and to justify a higher price than for other similar juices.

## Figures and Tables

**Table 1:** Fruit origin, cultivation method, and technological aspects of the eight commercial PJs.

Sample Code	Fruit Cultivation Method	Fruit Origin	Juice Production	Concentration Process	Stabilization Technology
ICPJ1	Conventional	Unknown	From concentrate	Under vacuum water evaporation	High-Temperature, Short-Time (HTST) Pasteurization
INCPJ2	Conventional	Italy	Not from concentrate		High-Pressure Processing (HPP)
SNCPJ3	Conventional	Italy	Not from concentrate		High-Temperature, Short-Time (HTST) Pasteurization
INCPJ4	Organic	Greece	Not from concentrate		High-Pressure Processing (HPP)
SCPJ5	Organic	Extra UE	From concentrate juice	Water evaporation at atmospheric pressure	High-Temperature, Short-Time (HTST) Pasteurization
SCPJ6	Organic	Extra UE	From concentrate	Water evaporation at atmospheric pressure	High-Temperature, Short-Time (HTST) Pasteurization
ICPJ7	Organic	Extra UE	From concentrate	Water evaporation at atmospheric pressure	High-Temperature, Short-Time (HTST) Pasteurization
INCPJ8	Organic	Italy	Not from concentrate		High-Temperature, Short-Time (HTST) Pasteurization

**Table 2:** Total phenols, tannins, and their ratios contained in the eight PJs.

Samples	Total Phenols (mg/L)	Tannins (mg/L)	Tannins/Total Phenols (%)
ICPJ1	2181.2 ± 9.8a	710.0 ± 8.8a	32.5%
INCPJ2	1632.0 ± 15.7b	394.8 ± 2.8b	24.2%
SNCPJ3	2326.1 ± 15.2c	694.0 ± 7.5c	29.8%
INCPJ4	2736.3 ± 8.7d	601.2 ± 1.6d	22.0%
SCPJ5	1379.9 ± 8.5e	520.9 ± 0.9e	37.7%
SCPJ6	2181.9 ± 31.6a	787.6 ± 5.4f	36.1%
ICPJ7	3748.8 ± 19.8f	895.2 ± 1.6g	23.9%
INCPJ8	2614.0 ± 16.5g	722.8 ± 3.6h	27.7%

The results are the mean of two different determinations ± standard deviation. Different lowercase letters (a–h) in the column indicate that the data are significantly different with respect to each other (*p* < 0.001).

**Table 3:** Phenolic composition (mg/L) of PJs.

Compounds	ICPJ1	INCPJ2	SNCPJ3	INCPJ4	SCPJ5	SCPJ6	ICPJ7	INCPJ8
Digalloyl-glucoside *	114.0 ± 3.3a	61.3 ± 2.9b	174.1 ± 5.3c	235.1 ± 6.4d	64.8 ± 1.5b	99.3 ± 0.9a	291.4 ± 5.3e	227.4 ± 0.5d
Punicalin a	313.7 ± 1.0a	16.6 ± 0.3b	315.4 ± 11.9a	530.5 ± 6.7c	206.3 ± 1.4d	357.5 ± 1.9e	799.0 ± 7.5f	314.2 ± 0.4a
Punicalin b	398.5 ± 3.5a	22.9 ± 0.3b	464.2 ± 5.7c	711.4 ± 5.5d	263.3 ± 0.3e	466.4 ± 4.2c	1042.5 ± 5.3f	413.7 ± 5.8a
α-Punicalagin	53.5 ± 2.1a	5.1 ± 0.3b	20.9 ± 0.8c	51.4 ± 0.4a	25.0 ± 0.3cd	0 ± 0	42.8 ± 2.1e	29.0 ± 1.4d
β-Punicalagin	123.8 ± 5.6a	0 ± 0	0 ± 0	20.5 ± 0.4b	0 ± 0	175.2 ± 1.1c	20.8 ± 0.5b	0 ± 0
Ellagic acid glucoside	31.5 ± 0.4a	16.6 ± 0.7b	30.0 ± 1.5ac	26.2 ± 0.1d	14.4 ± 0.5b	28.1 ± 0.0c	34.7 ± 0.3e	20.4 ± 0.5f
Valoneic acid dilactone	21.8 ± 0.8a	0 ± 0	12.4 ± 0.7bc	9.4 ± 0.6c	0 ± 0	15.6 ± 0.1b	73.2 ± 3.4d	25.3 ± 1.4a
Ellagic acid	45.7 ± 1.0a	28.2 ± 1.7b	30.1 ± 0.4b	41.3 ± 0.4c	22.1 ± 0.3d	37.9 ± 0.0c	69.8 ± 1.6e	40.8 ± 1.3c
Sum of phenols	1102.3 ± 7.8a	150.8 ± 3.5b	1047.1 ± 14.4c	1625.8 ± 10.9d	596.0 ± 2.2e	1179.9 ± 4.9f	2374.2 ± 11.5g	1070.8 ± 6.3ac

* The results are the mean of two different determinations ± standard deviation. Different lowercase letters (a–g) in a line indicate that the data are significantly different from each other (*p* < 0.001).

**Table 4:** Anthocyanins (mg/L) of PJs.

Compounds	ICPJ1	INCPJ2	SNCPJ3	INCPJ4	SCPJ5	SCPJ6	ICPJ7	INCPJ8
Delphinidin 3,5-diglucoside *	2.4 ± 0.0a	n.d.	23.4 ± 0.0b	13.6 ± 0.1c	0.5 ± 0.0d	0.7 ± 0.0e	0.3 ± 0.0d	85.0 ± 0.1f
Cyanidin 3,5-diglucoside	30.0 ± 0.0a	n.d.	29.7 ± 0.1b	14.7 ± 0.0c	1.1 ± 0.0d	1.7 ± 0.0e	0.5 ± 0.0f	65.6 ± 0.0g
Delphinidin 3-glucoside	0 ± 0	n.d.	9.2 ± 0.0a	12.8 ± 0.0b	0 ± 0	0.2 ± 0.0c	0 ± 0	62.5 ± 0.1d
Cyanidin 3-glucoside	15.1 ± 0.1a	n.d.	12.2 ± 0.0b	18.5 ± 0.0c	0 ± 0	0.3 ± 0.0d	0 ± 0	67.5 ± 0.0e
Sum of anthocyanins	47.7 ± 0.1a	n.d.	74.5 ± 0.1b	59.7 ± 0.1c	1.6 ± 0.0d	2.9 ± 0.0e	0.8 ± 0.0f	280.6 ± 0.1g

* The results are the mean of two different determinations ± standard deviation. Different lowercase letters (a–g) in line indicate that the data are significantly different from each other (*p* < 0.05).

**Table 5:** Antioxidant activity (µmol TE/mL) of PJs evaluated according to different methods.

Samples *	DPPH^•^	FRAP	ABTS
ICPJ1	43.3 ± 0.1ac	33.6 ± 1.6ac	48.83 ± 0.25a
INCPJ2	27.0 ± 0.7b	19.8 ± 0.1b	14.29 ± 0.4b
SNCPJ3	40.0 ± 3.4c	32.5 ± 1.0c	28.68 ± 1.16c
INCPJ4	49.7 ± 0.4a	39.7 ± 0.6d	31.56 ± 0.86d
SCPJ5	25.4 ± 0.7b	18.4 ± 0.2b	18.68 ± 0.34e
SCPJ6	49.9 ± 0.5a	37.4 ± 0.6ad	35.25 ± 0.06f
ICPJ7	69.1 ± 3.6d	56.6 ± 0.2e	46.7 ± 0.22a
INCPJ8	47.9 ± 0.4a	36.1 ± 0.3acd	34.64 ± 0.44f

* The results are the mean of two different determinations ± standard deviation. Different lowercase letters (a–f) in the column indicate that the data are significantly different from each other (*p* < 0.05). DPPH^•^: 2,2′-diphenyl-1-picrylhydrazyl; FRAP: ferric reducing antioxidant power; ABTS: 2,2′-azinobis-(3-ethylbenzothiazoline-6-sulphonate); TE: TROLOX equivalent.

**Table 6:** Organic acids (g/L) present in PJs.

Samples *	Malic Acid	Citric Acid	Total
ICPJ1	1.2 ± 0.1a	7.3 ± 0.0a	8.5 ± 0.1a
INCPJ2	1.6 ± 0.1ac	9.1 ± 0.0b	10.7 ± 0.1b
SNCPJ3	3.0 ± 0.1b	9.5 ± 0.0c	12.5 ± 0.1c
INCPJ4	3.1 ± 0.0b	11.0 ± 0.1d	14.1 ± 0.1d
SCPJ5	2.7 ± 0.1b	4.7 ± 0.1e	7.5 ± 0.1e
SCPJ6	2.1 ± 0.1d	8.3 ± 0.0f	10.4 ± 0.1b
ICPJ7	4.6 ± 0.2e	14.7 ± 0.0g	19.3 ± 0.2f
INCPJ8	1.9 ± 0.1cd	11.0 ± 0.0d	12.9 ± 0.1c

* The results are the mean of two different determinations ± standard deviation. Different lowercase letters (a–g) in within columns indicate that the data are significantly different from each other (*p* < 0.001).

**Table 7:** Odour thresholds (OT) of volatile compounds found in PJs [76,77,78,79,80,81,82,83,84,85,86,87,88].

Compounds	OT (µg/L)	Sensory Descriptor
Aldehydes		
Acetaldehyde	15–120 [76]; 110 [77]	Pungent, refreshing, green
2- Methylbutanal	1.5 [78]; 1.5–4.5 [79]; 10 [80]	Malt, mould, coffee, chocolate
3- Methylbutanal	0.5 [78]; 1.6 [80]; 4.6 [81]; 5.4 [77]	Malt
Benzaldehyde	350–2000 [79]; 990 [82]; 2000 [81]	Bitter, almond
3-Methyl-benzaldehyde	n.d.	Sweet, fruity, cherry
Alcohols		
Ethanol	25,000–900,000 [76]; 100,000 [83]	Alcoholic, ripe apple
2-Methyl-1-propanol	40,000 [82]	Ethereal, vinous
2-Methyl-3-buen-2-ol	n.d.	Herbaceous, earthy
1-Pentanol	5 [84]	Fruity
1-Hexanol	250 [83]; 1600 [77]; 2500 [84]	Banana, floral, grass
3-Hexen-1-ol	110(E)–910(Z) [77]; 200 [84]	Grass, green fruits, unripe banana
Phenylethyl alcohol	140–390 [79]	Floral, honey, sweet
Esters		
(Z)-3-Hexenyl acetate	13 [85]; 16 [77]	Green banana, fruity, floral
Methyl benzoate	0.5 [85]	Phenolic, almond, floral
Ketones		
Acetoin	800	Sweet, butter
Acids		
Acetic acid	26,000 [77]	Vinegar
2-Methylpropanoic acid	16,000–29,000 [79]	Sweet, cheese
2-Methylbutanoic acid	1580 [76]; 2200 [78]	Pungent, sour, cheese
Furans		
3-Furfural	8000 [84]	Bread, almonds, cooked potatoes
Acetyl furan	n.d.	Caramel, coffee, balsamic
5-Methylfurfural	2000 [81]	Sweet, bitter almonds
Tetrahydro-2-furanone	n.d.	Creamy oily fatty caramel
Terpenes		
4-terpineol	1200 [77]	Mould, dust
α-terpineol	280–350 [86]; 5000 [77]	Pine, citric, woody, floral
Eucalyptol	1.1 [85]; 1.3–12 [76]; 4.6 [87]	Eucalyptus
Menthone	170	Mint
Carvone	2.7–800 [88]; 20 [87]	Mint, liquorice

**Table 8:** Volatile compounds (µg/L).

Compounds	ICPJ1	INCPJ2	SNCPJ3	INCPJ4	SCPJ5	SCPJ6	ICPJ7	INCPJ8
Aldehydes *								
Acetaldehyde	5.5 ± 0.7a	11.8 ± 0.5b	27.6 ± 0.3c	10.4 ± 0.2b	6.6 ± 0.8a	4.3 ± 0.4ad	5.4 ± 0.2a	2.9 ± 0.3d
2- Methylbutanal	14.8 ± 1.5a	9.4 ± 1b	9.8 ± 1.4b	4.3 ± 0.3c	0 ± 0	3.8 ± 0c	n.d.	2.2 ± 0.3c
3- Methylbutanal	11.6 ± 1.3a	15.2 ± 0.5b	4.8 ± 0.2c	1.2 ± 0.1d	3.6 ± 0.7cd	4.9 ± 0.4cd	n.d.	n.d.
Benzaldehyde	12.3 ± 0.6a	11.1 ± 0.7ab	10.4 ± 0.3b	4.8 ± 0c	8.2 ± 0.3d	7.8 ± 0.3d	7.9 ± 0.3d	2.1 ± 0.1e
3-Methyl-benzaldehyde	4.4 ± 0.1ac	4.4 ± 0.1ac	3.5 ± 0.4b	4.8 ± 0.4ac	4.2 ± 0.2a	3.3 ± 0.3b	5.3 ± 0.4c	5 ± 0.1c
Sum of aldehydes	48.6 ± 2.2a	51.9 ± 1.4ab	56.1 ± 1.5b	25.5 ± 0.5c	22.6 ± 1.1cd	24.2 ± 0.8c	18.6 ± 0.5d	12.2 ± 0.4e
Alcohols								
Ethanol	15.4 ± 0.4a	1129.5 ± 26.9b	2589.3 ± 19.1c	1460.6 ± 81.6d	84.2 ± 6.6e	52 ± 1.2f	53.1 ± 5.1f	1198 ± 95b
2-Methyl-1-propanol	0 ± 0	2.1 ± 0.1a	1.7 ± 0b	0 ± 0	0.2 ± 0c	0.2 ± 0c	0 ± 0	0 ± 0
2-Methyl-3-buen-2-ol	13.9 ± 0.6a	0 ± 0	10.9 ± 0.4b	13.4 ± 0.7ab	4.5 ± 0.2c	6.1 ± 0.4c	26.6 ± 1.2d	18.6 ± 0.9e
1-Pentanol	0 ± 0	2.4 ± 0.1a	2.4 ± 0.1a	2.8 ± 0.1a	n.d.	0.4 ± 0b	0.5 ± 0b	4.1 ± 0.3c
1-Hexanol	0.1 ± 0a	38.1 ± 2.4b	28.1 ± 0c	30 ± 0.5c	0.1 ± 0a	0.1 ± 0a	0.1 ± 0a	7.5 ± 0.4d
3-Hexen-1-ol	n.d.	10.5 ± 0.4a	16.4 ± 0.7b	43.5 ± 0.8c	n.d.	n.d.	n.d.	3.3 ± 0d
Phenylethyl alcohol	0.4 ± 0a	0.1 ± 0b	0.3 ± 0c	0.3 ± 0c	0.2 ± 0d	0.2 ± 0d	0.5 ± 0e	0.1 ± 0b
Sum of alcohols	29.9 ± 0.7a	1182.7 ± 27b	2649.2 ± 19.1c	1550.6 ± 81.6d	89.2 ± 6.6e	58.9 ± 1.3f	80.8 ± 5.2e	1231.7 ± 95b
Esters								
(Z)-3-Hexenyl acetate	1.2 ± 0a	n.d.	3.7 ± 0.4b	n.d.	0.4 ± 0c	0.5 ± 0c	2.1 ± 0d	2.1 ± 0.2d
Methyl benzoate	10.7 ± 1a	9.4 ± 1a	10.1 ± 0.4a	8.5 ± 0.3a	9 ± 0.6a	9.2 ± 0.5a	10.2 ± 0.5a	9.5 ± 0.9a
Sum of esters	11.9 ± 1ac	9.4 ± 1b	13.9 ± 0.6c	8.5 ± 0.3b	9.4 ± 0.6b	9.7 ± 0.5b	12.3 ± 0.5ac	11.5 ± 0.9a
Ketones								
Acetoin	7.1 ± 0a	3 ± 0.2b	36.7 ± 1.8c	8.2 ± 0.4a	1.8 ± 0b	2.1 ± 0.1b	6.7 ± 0.1a	1.5 ± 0b
Acids								
Acetic acid	374.8 ± 37.7a	11.7 ± 1.1b	303.7 ± 23.2c	62 ± 5.4d	48.5 ± 2.3e	72.2 ± 12.5d	526.3 ± 39.4f	14.6 ± 1.1b
2-Methylpropanoic acid	30.9 ± 0.1a	1.2 ± 0.1b	9 ± 0.2c	9.8 ± 0.6c	8.1 ± 0.6c	14.1 ± 2d	36.4 ± 0.9e	8 ± 1.2c
2-Methylbutanoic acid	92 ± 0.3a	1.9 ± 0.2b	20.1 ± 0.3c	22.9 ± 1c	21.3 ± 1.4c	33 ± 5.1d	59.1 ± 0.1e	14.5 ± 1.4f
Sum of acids	497.8 ± 37.7a	14.7 ± 1.1b	332.8 ± 23.2c	94.7 ± 5.5d	77.8 ± 2.7d	119.3 ± 13.6e	621.8 ± 39.5f	37.1 ± 2.1g
Furans								
3-Furfural	404.1 ± 13.2a	n.d.	879.9 ± 63.3b	46.2 ± 0.8c	128 ± 6.2d	64.3 ± 2.9c	243.3 ± 9.4e	8.6 ± 0.6f
Acetyl furan	9.7 ± 0.3a	n.d.	11.3 ± 1b	9 ± 0.5a	3.7 ± 0.1c	3 ± 0.1c	8.6 ± 0.3a	0.8 ± 0d
5-Methylfurfural	7.7 ± 0.2a	n.d.	4.4 ± 0.3b	0.8 ± 0c	2.1 ± 0.1d	1.5 ± 0.1e	4 ± 0.2b	0.6 ± 0c
Tetrahydro-2-furanone	3.6 ± 0.3a	n.d.	0.8 ± 0.1b	1.2 ± 0.1c	1.3 ± 0c	1.7 ± 0.1c	5.3 ± 0d	0.5 ± 0b
Sum of furans	425.1 ± 13.2a	n.d.	896.4 ± 63.3b	57.2 ± 0.9c	135.1 ± 6.2d	70.5 ± 2.9c	261.1 ± 9.4d	10.5 ± 0.6e
Terpenes								
4-terpineol	n.d.	2.4 ± 0.1a	5.3 ± 0b	0.9 ± 0c	0 ± 0	0.6 ± 0.1c	0.9 ± 0.1c	2.2 ± 0.2a
α-terpineol	n.d.	6.4 ± 0.1a	11.7 ± 0.4b	9.1 ± 0.4c	1 ± 0d	0.8 ± 0d	0.9 ± 0.1d	5.3 ± 0.1a
Eucalyptol	n.d.	n.d.	n.d.	n.d.	n.d.	n.d.	n.d.	3.2 ± 0.3
Menthone	n.d.	2 ± 0.2a	0.2 ± 0b	n.d.	n.d.	0.2 ± 0b	n.d.	4.1 ± 0.4c
Carvone	n.d.	n.d.	0.2 ± 0a	0.1 ± 0a	n.d.	n.d.	n.d.	4.5 ± 0.2b
Sum of terpenes	n.d.	10.8 ± 0.3a	17.4 ± 0.4b	10.1 ± 0.4a	1 ± 0c	1.6 ± 0.1c	1.8 ± 0.1c	19.4 ± 0.6d
Sum of volatile compounds	1020.4 ± 40a	1272.5 ± 27.1b	4002.4 ± 70.2c	1754.8 ± 81.8d	336.9 ± 9.5e	286.3 ± 14f	1003.1 ± 40.9a	1323.8 ± 95b

* The results are the mean of two different determinations ± standard deviation. Different lowercase letters (a–g) in line indicate that the data are significantly different from each other (*p* < 0.05).

## Data Availability

Data are contained into the article and Supplementary Material.

## Supplementary Materials

The following are available online at https://www.mdpi.com/article/10.3390/antiox10091381/s1, Figure S1: Sensory evaluation of PJs: (a) Tasting room set up for the PJ sensory analysis; (b) the presentation of samples analysed in detail. Figure S2: Sensory analysis of the eight commercial PJs: the upper side spider plots (a) and (b) show the appearance and smell attributes of PJ samples, respectively. In the lower side, the spider plots (c) and (d) show the texture/mouthfeel and taste attributes of PJ samples, respectively. Table S1: Nutritional values of the eight commercial PJs. Table S2: Chemical and spectroscopic characteristics of phenolic compounds revealed by HPLC-DAD-ESI-Q-TOF analysis. Table S3: Sensory attributes list with definitions used for the sensory evaluation of PJs.
