# Supplementary material for: Chemical Composition, Antioxidant Activity, and Sensory Characterization of Commercial Pomegranate Juices
Source: Antioxidants (Basel). 2021 Aug 29;10(9):1381. doi: 10.3390/antiox10091381 (PMC8471094; doi:10.3390/antiox10091381)
Supplement: Supplementary file 1 [file antioxidants-10-01381-s001.zip › Table S1.pdf]

Table S1. Nutritional values of the eight commercial PJs.

Samples *	Fats (%)	Saturated fats (%)	Sugars (%)	Simple sugars (% of sugars)	Proteins (g)	Fibers (g)	Salt (g)
**ICPJ1**	<0.5	<0.1	14	14	<0.5	<0.5	0.02
**INCPJ2**	0.0	0.0	12.29	8.9	0	0	0
**SNCPJ3**	0.29	0.08	13.3	13.7	0.15	0.1	0.001
**INCPJ4**	1.2	0.3	13.7	12.3	1.9	2	<0.03
**SCPJ5**	<0.5	<0.1	12	12	<0.5	<0.5	<0.01
**SCPJ6**	<0.5	<0.1	11	9.4	<0.5	<0.5	<0.01
**ICPJ7**	<0.5	0.0	12	12	0	1.3	0.02
**INCPJ8**	2.0	0.1	16	16	0.5	2.2	0.0175

* All samples were no added sugars, acidifiers and preservatives.
