# Supplementary material for: Chemical Composition, Antioxidant Activity, and Sensory Characterization of Commercial Pomegranate Juices
Source: Antioxidants (Basel). 2021 Aug 29;10(9):1381. doi: 10.3390/antiox10091381 (PMC8471094; doi:10.3390/antiox10091381)
Supplement: Supplementary file 1 [file antioxidants-10-01381-s001.zip › Table S2.pdf]

Table S2. Chemical and spectroscopic characteristics of phenolic compounds revealed by HPLC-DAD-ESI-Q-TOF analysis.

Assignment	Molecular formula	Retention time (min)	UV wave lenght (nm)	[M-H] Calculated	[M-H] Expected	Score	Difference (ppm)
Di-galloyl-glucoside	$C_{20}H_{20}O_{14}$	2.69	275	483.078	483.078	99.66	-0.66
Punicalin *a*	$C_{34}H_{22}O_{22}$	2.81	256;374	781.053	781.054	99.88	0.61
Punicalin *b*	$C_{34}H_{22}O_{23}$	2.90	256;374	781.053	781.053	99.09	0.30
α-Punicalagin	$C_{48}H_{28}O_{30}$	3.46	260;378	1083.059	1083.056	91.44	-1.38
β-Punicalagin	$C_{48}H_{28}O_{31}$	3.97	260;378	1083.059	1083.059	98.09	-0.56
Valoneic acid dilactone	$C_{21}H_{10}O_{13}$	11.47	269;370	469.005	469.005	99.42	-0.49
Ellagic acid glucoside	$C_{20}H_{16}O_{13}$	11.64	256;365	463.052	463.052	99.59	-0.38
Ellagic acid	$C_{14}H_{6}O_{8}$	15.26	254;369	300.999	300.999	99.36	0.69
