# Supplementary material for: Chemical Composition, Antioxidant Activity, and Sensory Characterization of Commercial Pomegranate Juices
Source: Antioxidants (Basel). 2021 Aug 29;10(9):1381. doi: 10.3390/antiox10091381 (PMC8471094; doi:10.3390/antiox10091381)
Supplement: Supplementary file 1 [file antioxidants-10-01381-s001.zip › Table S3.pdf]

**Table S3.** Sensory attributes list with definitions used for the sensory evaluation of PJs.

| | Attributes | Definitions |
|---|---|---|
| **Appearance** | Brick-red colour | Colour which might change from the brick red colour to the more or less intense brown colour. |
| | Purple-red colour | Colour which might change from red colour to more or less intense purple colour. |
| | Cloudy | Opalescence; difficulty to see trough a transparent glass. |
| **Smell** | Pomegranate fruit | Sour, sweet and fruity aromas. These aromas remind the assessor to a combination of grape, redberry, and other berries such as blackberries, cherries, currants, raspberries. Vegetable notes like beets and carrots might be also present. |
| | Fresh fruit | General term for describing sweet, floral and fruity aromas, correlated to a just extracted fruit. |
| | Tomato | Aroma associated to the tomato fruit. |
| | Berry | Sweet, slightly sour aromas related to ripe berries, such as raspberries, blueberries, blackberries, currants. |
| | Cherry | Sour, fruity and slightly bitter aromas commonly related to the cherries. |
| | Cooked apple | Sweet slightly fruity and floral aroma, commonly related to a processed apple juice and cooked apples. |
| | Plum | Sweet aromas, typical of dried plum. |
| | Floral | Sweet and perfumed perception reminding to the flowers. |
| | Acetic acid | Odour associated to the acetic acid. |
| | Vegetable-herbal | Typical aroma of just cut grape vine and stem. |
| | Brown spices (star anise, clove) | Sweet, refreshing and spicy aromas associated with star anise or clove. |
| | Wilted fruit | Sweet honey or caramel-like aromas commonly associated with wilted fruits, such as raisins. |
| | Candies | Sweet aroma often associated with processed essential oils and usually found in products such as gummy candies. |
| | Caramel | Sweet aroma that may include notes of character identified as caramel and commonly associated with honey, brown sugar, caramel or other products that have undergone non-enzymatic browning and Maillard reaction. |
| | Honey | Sweet, slightly spicy aroma associated with honey. |
| | Beet | Earthy and slightly sweet aromas commonly associated with canned or cooked red beets. |
| | Mushroom/Earthy | Aroma similar to moist soil, characteristic of cellars or similar. |
| | Metallic | Flavour of a slightly oxidized metal, such as iron, copper and / or a silver teaspoon. |
| | Cooked | Sweet aroma associated with that of caramel and honey. |
| **Texture/Mouthfeel** | Effervescence | A feeling of an increased sensation on the tongue that may be due to intense carbonation or other causes. Evaluate during first 3–5 s after sample is placed in the mouth. |
| | Tamarind | Aroma associated with tamarind, that is sour, very fruity and refreshing. |
| | Alcohol | Intense fruity aroma similar to ethanol, aromatic associated with alcohol from fermented grape (red wine). |
| | Metallic | A mouthfeel and aromatics associated "tin" cans, iron copper, or oxidized silver. |
| | Dryness | A sensation of abrasion and drying of the surface of the teeth. |
| | Homogeneity | Presence of fragments found in the mouth. |
| | Woody | Aroma associated with chopped wood. |
| | Pungent | A sharp, physically penetrating sensation in the nasal cavity. |
| | Astringency | Dry puckering mouthfeel associate with tea leaves. |
| **Taste** | Sweet | Taste associated with a sucrose solution. |
| | Sour | Taste associated with an acetic acid solution. |
| | Bitter | Taste associated with caffeine or quinine. |
