# Supplementary figures and images for: Chemical Composition, Antioxidant Activity, and Sensory Characterization of Commercial Pomegranate Juices
Source: Antioxidants (Basel). 2021 Aug 29;10(9):1381. doi: 10.3390/antiox10091381 (PMC8471094; doi:10.3390/antiox10091381)

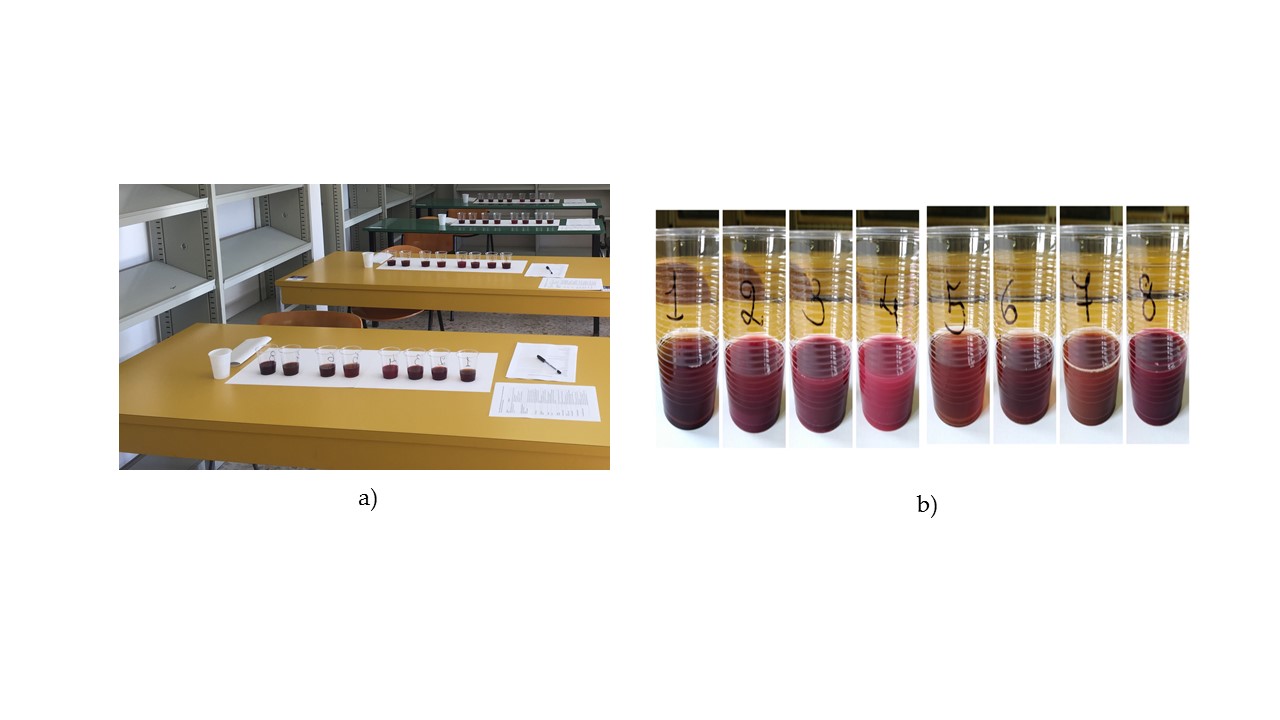

Supplement: Supplementary file 1 [file antioxidants-10-01381-s001.zip › Figure S1.jpg]

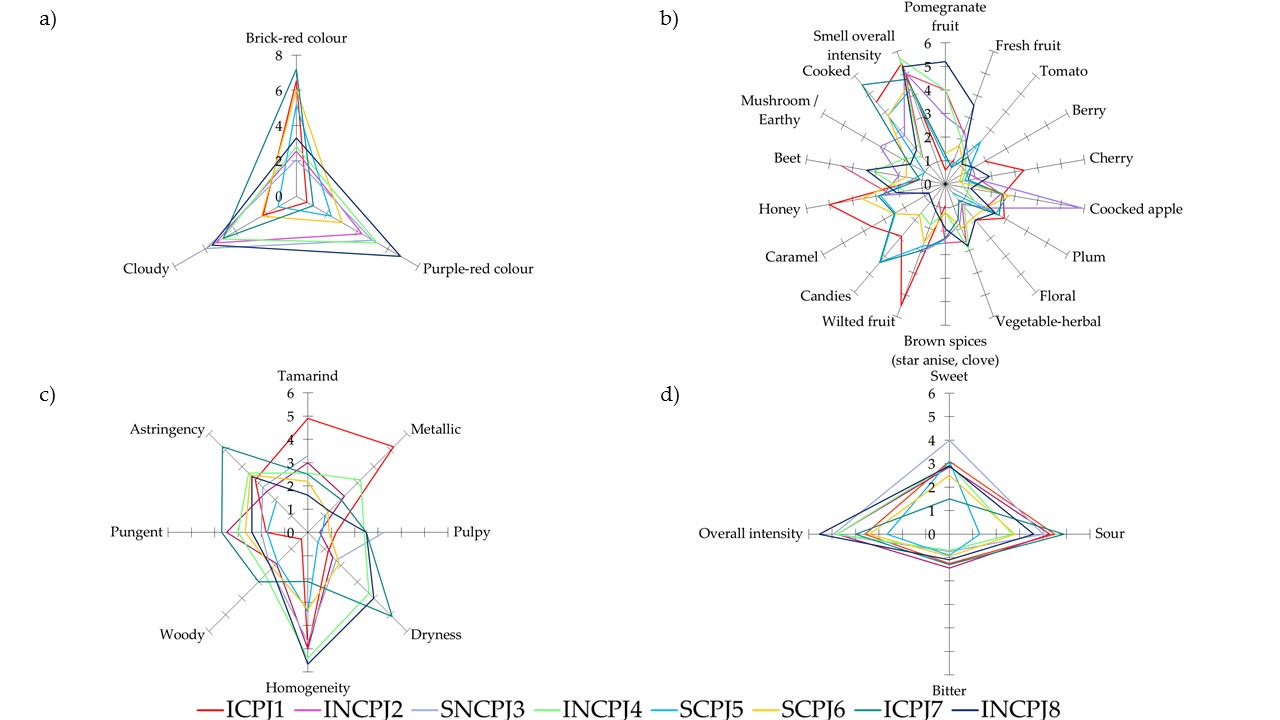

Supplement: Supplementary file 1 [file antioxidants-10-01381-s001.zip › Figure S2.jpg]
